# From Lexical Tone to Lexical Stress: A Cross-Language Mediation Model for Cantonese Children Learning English as a Second Language

**DOI:** 10.3389/fpsyg.2017.00492

**Published:** 2017-03-31

**Authors:** William Choi, Xiuli Tong, Leher Singh

**Affiliations:** ^1^Division of Speech and Hearing Sciences, The University of Hong KongHong Kong, Hong Kong; ^2^Department of Psychology, National University of SingaporeSingapore, Singapore

**Keywords:** lexical prosody, tone sensitivity, stress sensitivity, prosodic transfer, ESL

## Abstract

This study investigated how Cantonese lexical tone sensitivity contributed to English lexical stress sensitivity among Cantonese children who learned English as a second language (ESL). Five-hundred-and-sixteen second-to-third grade Cantonese ESL children were tested on their Cantonese lexical tone sensitivity, English lexical stress sensitivity, general auditory sensitivity, and working memory. Structural equation modeling revealed that Cantonese lexical tone sensitivity contributed to English lexical stress sensitivity both directly, and indirectly through the mediation of general auditory sensitivity, in which the direct pathway had a larger relative contribution to English lexical stress sensitivity than the indirect pathway. These results suggest that the tone-stress association might be accounted for by joint phonological and acoustic processes that underlie lexical tone and lexical stress perception.

## Introduction

**FIGURE 1:**
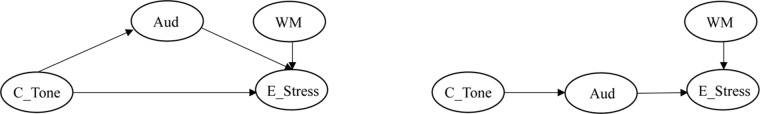
**The partial mediation model (Left) and the full mediation model (Right) for the relation between Cantonese lexical tone sensitivity and English lexical stress sensitivity.** C_Tone = Cantonese lexical tone sensitivity; E_Stress = English lexical stress sensitivity; Aud = General auditory sensitivity; WM = Working memory.

Suprasegmental information such as lexical tones and lexical stress surface with high frequency across languages of the world (Gussenhoven, 2004) and serve as two primary means by which languages contrast words using suprasegmental cues (e.g., Cutler and Chen, 1997). Relative to the segmental dimension, e.g., consonants and vowels (e.g., Chien et al., 2008; Keung and Ho, 2009; Wang et al., 2009), there is a comparative paucity of research on the extent to which sensitivity to suprasegmental cues generalize across languages over the course of first language (L1) and second language (L2) development. Noticeably, there is emerging research demonstrating lexical prosodic transfer, a process through which adults and children who learn English as second language (ESL) capitalize on similarities in the structure of lexical tones and lexical stress in a way that allows them to harness their perceptual sensitivity to L1 lexical tones in the service of L2 English lexical stress perception (e.g., Nguyen et al., 2008; Wang, 2008; Yu and Andruski, 2010; Tong et al., 2015a, 2016). In the context of Cantonese ESL children, a previous study reported that Cantonese lexical tone sensitivity contributed to English lexical stress sensitivity (Tong et al., 2016). Little is known, however, on *how* Cantonese lexical tone sensitivity contributes to English lexical stress sensitivity among Cantonese ESL children. More specifically, attested links between Cantonese lexical tone perception and English lexical stress perception could be direct, as in Tong and colleagues’ study, or they could be mediated by other candidate processes. In addition to its theoretical significance, this question has substantial practical significance given the role of English lexical stress sensitivity in English word reading and English reading comprehension not only amongst English speaking children (e.g., Arciuli et al., 2010; Holliman et al., 2010) but also amongst Cantonese ESL children (e.g., Choi et al., 2016a). To further unpack the contribution of Cantonese lexical tone sensitivity to English lexical stress sensitivity in Cantonese ESL children, we proposed and evaluated two structural equation models, i.e., a full model and a nested model (**Figure 1**). The full model consisted of two possible pathways through which Cantonese lexical tone sensitivity might contribute to English lexical stress sensitivity, i.e., the direct pathway and an indirect pathway with the mediation of general auditory sensitivity. The nested model was nested within the full model, and consisted only of the indirect pathway.

### Contribution of Cantonese Lexical Tone Sensitivity to English Lexical Stress Sensitivity: The Direct Pathway

We hypothesize that there is a direct pathway from Cantonese lexical tone sensitivity to English lexical stress sensitivity. One theoretical foundation of this hypothesis lies in the structural and functional similarities between Cantonese lexical tone and English lexical stress. In terms of their composition, the assignment of lexical tone and of lexical stress has a common gestural basis in that they both involve modulating the rate of laryngeal vibration. The primary consequence of this is variation in the fundamental frequency of speech (vocal pitch). Although theoreticians have sometimes likened the way in which vocal pitch is manipulated to convey both tone and stress (e.g., Chrabaszcz et al., 2014), there are important differences in how tone and stress are assigned. In English, stress consists primarily of variation in vocal pitch, duration and intensity (Fry, 1958). In contrast to stress in English, lexical tone in Cantonese involves the use of vocal pitch (as well as amplitude, duration and other spectral factors) to distinguish lexical items at the syllable level and a tone is assigned to every syllable (Chao, 1968). Tones may change in a sentential context although each tone maintains its basic form even in a multi-word context. Stress and tone further differ in their relative scope of influence in the two languages investigated in the present study: lexical tones distinguish a broad set of words in Cantonese whereas lexical stress distinguishes a small set of words in English.

Tone and stress are therefore similar in structure, both being driven by a similar set of acoustic concomitants. In terms of the magnitude of fundamental frequency variation, there is a high correspondence between values assigned to tone bearing syllables in tone languages and to stress bearing syllables in stress languages, although the rate of fluctuation in fundamental frequency is higher in tone marking than in stress marking (Eady, 1982). However, subtle variations aside, tone and stress marking are compositionally similar. They are also functionally similar, both distinguishing minimally contrastive forms. Using Cantonese as an example, /ma/ in a high level tone /ma1/ means ‘mother,’ while /ma/ in a high rising tone /ma2/ means ‘horse.’ Similarly, in English, the words “CONtent” /‘kantεnt/ and “conTENT” /kən‘tεnt/ vary minimally by stress placement and represent different lexical items.

Theoretical support for links between stress and tone comes from phonological assimilation (Kuhl, 1991; Best, 1995; Flege, 1995; Best and Tyler, 2007). Specifically, models of non-native speech perception generally posit that L2 sounds are categorically perceived within, or assimilated to the L1 sound classes among L2 learners (see Cutler, 2012 for a detailed review). A parallel claim in the suprasegmental dimension is suggested by previous studies that have demonstrated the “tonalization” of English lexical stress, in which the English lexical stress were perceived as high tones by Cantonese ESL listeners (Luke, 2000; Lai, 2003; Chan, 2007). Furthermore, Tong et al. (2015a) proposed that lexical tone and lexical stress learning gave rise to the formation of a “general suprasegmental prototype” common to Cantonese lexical tone and English lexical stress, motivated by the acoustic similarity of Cantonese lexical tone and English lexical stress, and the association between Cantonese lexical tone sensitivity and English lexical stress sensitivity. Collectively, these studies suggest the possibility that Cantonese ESL listeners assimilated English lexical stress into their native tonal system, giving rise to a possible direct contribution of Cantonese lexical tone sensitivity to English lexical stress sensitivity.

### Contribution of Cantonese Lexical Tone Sensitivity to English Lexical Stress Sensitivity: The Indirect Pathway

Tone-stress links could be mediated by language-general auditory sensitivities to acoustic-phonetic variation. Notably, Wang et al. (2005) proposed a relation between Mandarin lexical tone sensitivity and general auditory sensitivity. Similarly, Zhang and McBride-Chang (2010) raised the possibility that auditory sensitivity to rhythmic changes influenced sensitivity to Cantonese lexical tones among Cantonese children. The purported relation between general auditory sensitivity and lexical tone sensitivity was later examined among Cantonese children by means of structural equation modeling (Zhang and McBride-Chang, 2014). In Zhang and McBride-Chang’s best-fit model, general auditory sensitivity was associated with Cantonese lexical tone perception. Specifically, the frequency discrimination tasks and the amplitude modulation task, presumably reflecting temporal and rhythmic sensitivities, respectively, correlated significantly with Cantonese lexical tone perception. This might suggest that tone language speakers harness lower level auditory perceptual sensitivity in the service of tone perception. The relation between general auditory sensitivity and suprasegmental speech perception may as well extend to English lexical stress. As described above, English lexical stress patterns are acoustical variations of fundamental frequency, duration, intensity and formant frequency of one syllable relative to another (e.g., Chrabaszcz et al., 2014). This raises the possibility that sensitivity to English lexical stress is associated with general auditory sensitivity as well as the further possibility that commonalities in lexical tone and lexical stress perception in tone language learners may be mediated by general auditory sensitivity.

Our proposition of the general auditory sensitivity mediated pathway is suggested by previous studies of lexical prosodic transfer from lexical tone to English lexical stress at the acoustic level (Nguyen et al., 2008; Wang, 2008; Yu and Andruski, 2010). In a series of tasks designed to measure lexical stress discrimination, identification and matching, the studies set out to explore the acoustic cues attended to by ESL adult listeners in perceiving English lexical stress. In a lexical stress identification task, Wang systematically manipulated the fundamental frequency, duration and intensity cues of the English lexical stress stimuli. As reflected by the reliance scores to the specific acoustic cues (Wang, 2008, p. 113 for the computation of reliance scores), Mandarin ESL listeners showed a greater reliance on fundamental frequency, and lesser reliance on duration and intensity relative to native English listeners when identifying lexical stress position. In a later study, Yu and Andruski reported that Mandarin ESL listeners consistently relied on fundamental frequency for identifying trochaic and iambic stress patterns in English real words, pseudowords and hums, and treated duration only as a secondary cue when identifying iambic stress patterns under the pseudoword condition. Native English listeners, on the other hand, relied on a more varied set of acoustic cues, including fundamental frequency, duration, intensity and vowel quality, across different stress patterns and linguistic conditions. Similar results have been reported among Vietnamese ESL listeners, who attended to fundamental frequency, but not duration in a stress matching task. Subtle variations aside, the above studies offer converging evidence that that L1 tonal listeners avail of acoustic cues to tone perception, specifically fundamental frequency, in order to process lexical stress. This suggests that ESL adults with L1 tone language experience draw on the acoustic commonalities across their languages to streamline processing of suprasegmental cues.

The current study set out to investigate how Cantonese lexical tone sensitivity contributed to English lexical stress sensitivity in Cantonese children learning English as a second language (ESL). Specifically, we tested how Cantonese lexical tone sensitivity contributed to English lexical stress sensitivity, but not the other way round. Our proposition was based on non-native speech perception models, e.g., the Speech Learning Model (Flege, 1995), which posited that L2 speech perception was susceptible to L1 influence (Cutler, 2012 for a detailed review). Additionally, it has been suggested the direction of cross-language transfer is predominantly governed by language proficiency, in which transfer occurs from the more proficient language to a less proficient language (e.g., Hernandez et al., 1994; Zhang et al., 2010). In the current study, the Cantonese ESL children were sequential bilinguals who were more proficient in Cantonese than English, and they had been actively developing their Cantonese tonal system since birth even though they had not yet reached adult-like performance (Ciocca and Lui, 2003). Based on the above, it was conceivable that the skills involved in Cantonese lexical tone perception were likely to be drawn upon to scaffold the sensitivity to L2 English lexical stress, instead of the other way around. In order to determine the pathways underlying the relationship, we investigated whether the contribution was direct or mediated by general auditory sensitivity, or both. We have addressed these questions by means of structural equation modeling given that it is a well-established statistical method for mediation analysis. Different from traditional regression analysis which is suited to evaluating single regression equations, structural equation modeling allows for the simultaneous evaluation of a system of regression equations essential for mediation analysis (Nachtigall et al., 2003; MacKinnon et al., 2007). To illustrate, **Figure 1** (Left) depicts the system of regression equations under investigation, specifically, Cantonese lexical tone sensitivity has an effect on general auditory sensitivity, general auditory sensitivity has an effect on English lexical stress sensitivity, and Cantonese lexical tone sensitivity also has an effect on English lexical stress sensitivity. Critically, structural equation modeling allows the same variable, i.e., general auditory sensitivity to represent a regressant in one equation (Cantonese lexical tone sensitivity to general auditory sensitivity) and a regressor in another equation (general auditory sensitivity to English lexical stress sensitivity), which crucially informs a mediation analysis.

To examine the research questions, we proposed two models: a partial mediation model consisting of both direct (Cantonese lexical tone sensitivity to English lexical stress sensitivity) and auditory-mediated pathways (Cantonese lexical tone sensitivity to general auditory sensitivity to English lexical stress sensitivity), and a full mediation model that only includes the auditory-mediated pathway (**Figure 1**). As for the latent variables, we assessed second-to-third grade Cantonese ESL children on a range of abilities, including Cantonese lexical tone sensitivity, English lexical stress sensitivity, general auditory sensitivity and working memory. As shown in **Figure 1**, we included working memory as a control variable to control for the variance in English lexical stress sensitivity as it had been found to relate to suprasegmental speech perception (Mattys and Samuel, 2000).

## Materials and Methods

### Participants

A sample of 516 (276 boys and 240 girls) second-to-third grade Cantonese ESL children was recruited from primary schools in Hong Kong. The mean age of the participants was 8 years and 5 months (*SD* = 7.81 months). We chose to study this age on account of prior research showing that by this age, children would have fully acquired all six contrastive tones (Ciocca and Lui, 2003) and stress (Tong et al., 2015a). All children were native Cantonese speakers and L2 English learners and were born to Cantonese speaking parents. They had all learned English as of Grade 1. Thus, all children had been learning English for a minimum of 2–3 years. All children were raised in a Cantonese-speaking environment and had learned and spoken Cantonese since birth.

### Materials and Procedure

#### Cantonese Lexical Tone Sensitivity

An odd-one-out tone discrimination task (Tong et al., 2014, 2016; Choi et al., 2016a,b) was modified to assess children’s sensitivity to Cantonese lexical tones. There were 48 trials, each consisting of three real Cantonese monosyllabic words, with one tone differed from the others (e.g., /sɪŋ1/, /sa1/, /sɐu2/). There were all together 144 words. To ensure the words were familiar to the children, the Cantonese words we selected were high frequency words representing common objects or concepts. They were matched approximately on ratings of familiarity and syllabic structure, i.e., either CV or CVC. The pilot testing showed that 7 years old Cantonese children were able to understand the meanings of the presented words.

Each trial presented one of the eight minimum possible tone contrasts, i.e., mid level-low level, high rising-low rising, high level-mid level, high level-low level, low rising-low level, low falling-low level, low falling-low rising and high level-high rising, as in previous studies of tone perception in children of a similar age (e.g., Ciocca and Lui, 2003; Tong et al., 2014, 2016; Choi et al., 2016a,b). There were six repetitions for each tone contrast in the whole test.

In each trial during the testing, three Cantonese real words (e.g., /sɪŋ1/, /sa1/, /sɐu2/) were presented audibly via an amplification system to the children, with an inter-stimulus interval of 400ms. The positions of the target word (e.g., /sɐu2/) in the word sequence (e.g., /sɪŋ1/, /sa1/, /sɐu2/) were counterbalanced across trials. The children selected the word they identified as carrying a different lexical tone from the other two words by indicating on the testing booklet the position of the word. Prior to the testing, three practice trials with corrective feedback were given to ensure the children’s full understanding of the instruction of the test. All participants reported that they heard the Cantonese words clearly, and understood the task requirements, and responded correctly in the practice trials. The number of correct responses was tallied out of the 48 trials, and summed to yield an accuracy rate for each participant.

#### English Lexical Stress Sensitivity

A “DEEdee” task (e.g., Whalley and Hansen, 2006; Goswami et al., 2010) was adopted to assess children’s sensitivity to stress patterning in spoken English. This task has successfully assessed sensitivity to stress patterning among second-to-third grade Cantonese leaners of English (Choi et al., 2016b). There were two practice items and 18 test items. All test items were pre-recorded items and consisted of highly familiar names or titles of children’s books converted to a reiterative syllable “dee.” For example, the phrase “aLAddin” was replaced with three synthesized tokens “deeDEEdee” (stressed DEE syllable flanked by two unstressed dee syllables). In each trial, children were audibly presented with the spoken phrase “aLAddin,” followed by the two DEEdee phrases, e.g., “deeDEEdee”and “DEEdeedee.” The children then chose a match to the spoken phrase, by indicating on the testing booklet whether the match was the “first” or the “second” DEEdee phrase. As in a previous study (Choi et al., 2016b), the English learning Cantonese children reported that they were familiar with the English words presented. Response accuracy was logged for each child out of 18 trials and summed to yield an accuracy rate.

#### General Auditory Sensitivity

We adopted the beat perception in music task (Goswami et al., 2013) to measure children’s general auditory sensitivity. This task adopted a forced choice paradigm and each trial consisted of two series of musical notes each having a pulse rate of 500 ms. The numbers of “same” and “different” trails were the identical, and the order of presentation of these two types of trials was pseudorandomized. In different trials, the two series of musical notes exhibited metrical changes in the accented notes. For example, in a different trial, the accented notes in one sequence might exhibit a 100 ms or 166 ms delay in rhythmic structure. Children indicated on the testing booklet whether the two stimuli presented were the same or different. In total, there were 24 trials preceded by two practice trials.

#### Working Memory

A serial-order reconstruction task adapted from Majerus et al. (2006) was used as a measure of working memory. Short-term retention for order information was probed in this task. The task was presented as a game, in which children heard sequences of animal names (lion, cat, dog, cock, bear, wolf, and monkey) in Cantonese with increasing length from 3 to 7 names. All animal names were common vocabularies in Cantonese, and were all monosyllabic. Children reconstructed the order of presentation of the animals by putting a digit (1–7) in the boxes under the animals’ pictures. The maximum possible number of correct trials in this task was 10.

## Results

Descriptive statistics for the results of all tasks are summarized in **Table 1**. Of particular interest were correlations between Cantonese lexical tone sensitivity, English lexical stress sensitivity and general auditory sensitivity, all of which were significant (*ps* < 0.01). These correlations suggest that these variables share common variance required for structural equation modeling.

**Table 1:** Means, Standard Deviations, Reliabilities, and Inter-correlations of All Variables

Variables (maximum possible score)	1	2	3	4
(1) Cantonese lexical tone sensitivity (48)	–			
(2) English lexical stress sensitivity (18)	0.33^∗∗∗^	–		
(3) General auditory sensitivity (24)	0.27^∗∗∗^	0.22^∗∗∗^	–	
(4) Working memory (10)	0.18^∗∗∗^	0.14^∗∗^	0.19^∗∗∗^	–
*Mean*	24.41	11.06	14.97	6.68
*SD*	7.77	2.82	3.40	1.62
*Reliability*	0.82	0.50	0.55	0.57


*N* = 516; ^∗∗∗^*p* < 0.001; ^∗∗^*p* < 0.01.

### Testing Direct and Indirect Contributions of Cantonese Lexical Tone Sensitivity to English Lexical Stress Sensitivity: Nested Models Comparisons

Latent variable structural equation modeling of the covariances matrix was conducted with LISREL 8.80 (Joreskog and Sorbom, 2007). The four latent variables, i.e., Cantonese lexical tone sensitivity, English lexical stress sensitivity, general auditory sensitivity and working memory were modeled with the Cantonese lexical tone discrimination task, DEEdee task, beat perception in music task and animal task, respectively (**Figure 1**).

The partial mediation model and full mediation models were nested models as the latter was derived from the former by fixing the parameter (direct effect from Cantonese lexical tone sensitivity to English lexical stress sensitivity) to zero. Given that the differences in chi-square values between these two nested models are chi-square distributed with degrees of freedom equivalent to the differences between degree of freedom between these two models (Steiger et al., 1985), we used a chi-square different test to determine which model can better explain the relation between Cantonese lexical tone sensitivity and English lexical stress sensitivity.

The chi-square difference between the partial mediation model and the full mediation model was significant, Δ*χ^2^* (1, *N* = 512) = 31.19, *p* < 0.001. According to Schermelleh-Engel et al. (2003), a significant chi-square difference indicated that the null hypothesis of equal fit for the partial and full mediation models was rejected, reflecting that the two models did not fit the data equally well – in such a case, the less restrictive model with smaller chi-square was preferable because it fitted significantly better than the more restrictive model with larger chi-square. Thus, the less restricted partial mediation model, which had a smaller chi-square, *χ^2^* (7, *N* = 512) = 11.83, than the full mediation model, *χ^2^* (8, *N* = 512) = 43.02, was preferred.

We evaluated the goodness of fit of the data to the partial mediation model with several goodness of fit indices, i.e., Chi-square, comparative fit index (CFI), normed fit index (NFI), non-normed fit index (NNFI), and root-mean-square-error of approximation (RMSEA). According to Hu and Bentler (1999), a value of 0.95 or above for CFI, NFI, and NNFI, and a value less than 0.06 for RMSEA denote a good fit model. The partial mediation model fit the data well, *χ^2^* (7, *N* = 512) = 11.83, CFI = 0.98, NFI = 0.96, NNFI = 0.97, RMSEA < 0.06, predicting 14% of variance in English lexical stress sensitivity.

**FIGURE 2:**
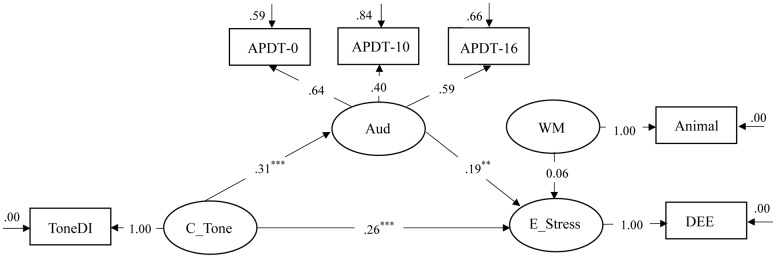
**Best fit model (partial mediation model) for the relation between Cantonese lexical tone sensitivity and English lexical stress sensitivity.** C_Tone = Cantonese lexical tone sensitivity; E_Stress = English lexical stress sensitivity; Aud = General auditory sensitivity; WM = Working memory; ToneDI = Cantonese lexical tone discrimination task; DEE = English lexical stress perception task; APPT-0 = Beat perception in music task (0 ms delay); APPT-100 = Beat perception in music task (100 ms delay); APPT-166 = Beat perception in music task (166 ms delay); Animal = Working memory task. The numerical values represent the standardized factor loadings. ^∗∗∗^*p* < 0.001; ^∗∗^*p* < 0.01.

Next, we evaluated the significance of the structural paths based on the *z* value associated with the unstandardized estimates of the path weights (Bentler, 2006). According to Bentler, a value of 1.96 or above for the *z* value indicates that the pathway is significant. In the partial mediation model, both direct and auditory-mediated pathways were significant, *ps* < 0.01 (**Figure 2**). These results suggest that Cantonese lexical tone sensitivity contributed to English lexical stress sensitivity both directly and mediated through general auditory sensitivity.

### Relative Contributions of the Direct and Auditory-Mediated Pathways

**FIGURE 3:**
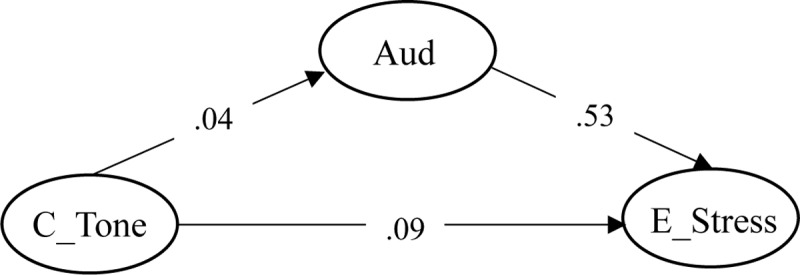
**Direct and auditory-mediated pathways through which Cantonese lexical tone sensitivity contributes to English lexical stress sensitivity.** Numerical values represent the coefficients of their designated routes. C_Tone = Cantonese lexical tone sensitivity; E_Stress = English lexical stress sensitivity; Aud = General auditory sensitivity.

We examined the relative contributions of the direct and indirect pathways by examining differences in the product coefficients of the two pathways (MacKinnon et al., 2007). In the partial mediation model, the product coefficients of the direct and indirect pathways were 0.09 and 0.02 (0.04 × 0.53), respectively (**Figure 3**). Thus, the coefficient difference of the two pathways was 0.09–0.02 = 0.07. With reference to MacKinnon and colleagues, a positive value of the coefficient difference indicates that the direct pathway has a larger relative contribution to the relationship between lexical tone and lexical stress perception than the indirect pathway.

## Discussion

The current study set out to investigate how Cantonese lexical tone sensitivity contributed to English lexical stress sensitivity within second-to-third grade Cantonese ESL children. Results indicate that Cantonese lexical tone sensitivity contributed to English lexical stress sensitivity both directly, and indirectly through the mediation of general auditory sensitivity. In terms of relative contribution, Cantonese lexical tone sensitivity made a larger direct contribution than indirect contribution to English lexical stress sensitivity.

Consistent with a previous study (Tong et al., 2016), we have shown the contribution of Cantonese lexical tone sensitivity to English lexical stress sensitivity among Cantonese ESL children. This finding has extended previous studies on L1 and L2 segmental dimension (Comeau et al., 1999; Chien et al., 2008; Keung and Ho, 2009), by demonstrating that the contribution of L1 to L2 phonological skills of Cantonese ESL children is also evident at the suprasegmental dimension. Placed in the context of previous studies on adult suprasegmental speech perception (Nguyen et al., 2008; Wang, 2008; Yu and Andruski, 2010), the results suggest that Cantonese ESL children are able to exploit common sources of phonological variation across languages, and the perceptual operations underlying L1 lexical tone perception might be recruited in service of L2 English lexical stress perception, consistent with the predictions of the non-native speech perception models, e.g., the Speech Learning Model (Flege, 1995). The Cantonese ESL children therefore appear to profit from cross-language commonalities by virtue of the finding that sensitivity to suprasegmental variation in one language facilitates prosodic sensitivity in the other language. Furthermore, the current study has further extended Tong and colleagues’ study by uncovering the underlying pathways through which Cantonese lexical tone sensitivity contributes to English lexical stress sensitivity. In particular, we found that Cantonese lexical tone sensitivity contributed to English lexical stress sensitivity through two pathways – the indirect pathway involving the mediation of general auditory sensitivity, and the direct pathway without the mediation of general auditory sensitivity.

### Direct Contribution from Cantonese Lexical Tone Sensitivity to English Lexical Stress Sensitivity

The direct pathway through which Cantonese lexical tone sensitivity contributed to English lexical stress sensitivity might be accounted for by joint phonological processes that underlie lexical tone and lexical stress perception. One possible shared component may be the ability to extract suprasegmental phonological information from full-spectral speech that includes segmental variation. Specifically, Cantonese lexical tones and English lexical stress are both instantiated on segments, most centrally, on the vowel (Cutler and Chen, 1997). In the Cantonese lexical tone discrimination task, which required children to integrate segmental variation and identify the odd tone, children had to extract tonal information from speech in order to compare the lexical tones of the target and distractors. Likewise, in the English stress perception task, children were required to identify reiterative stress patterns corresponding to that of a real word e.g., “deeDEEdee” for “aLAddin.” Similarly, children had to extract the lexical stress pattern from the speech in order to match the reiterative lexical stress pattern with that of the real words. Each of these abilities involved extracting suprasegmental variation from full-spectral input and applying this variation to a new word. This account is in line with previous neurophysiological (Choi et al., 2017) and behavioral studies of tone perception (Repp and Lin, 1990; Lee and Nusbaum, 1993; Tong et al., 2008, 2014) which demonstrated that segmental and suprasegmental information were processed integrally rather than independently, suggesting that speech arrives at our senses as an integrated signal that has to be segregated in response to task demands. The ability to segregate the signal in this way and to extract and re-apply suprasegmental phonological information from full-spectral speech may underlie the direct contribution of Cantonese lexical tones sensitivity to English lexical stress sensitivity.

Another possible shared phonological component might be the phonological encoding of suprasegmental information. To date, evidence suggests that lexical tones (Singh et al., 2015; Tong et al., 2015b; Choi et al., 2016a; Luo et al., 2016) and lexical stress (Ashby and Clifton, 2005; Arciuli et al., 2010; Goswami et al., 2013) are encoded as essential components of phonological representations in Chinese and English, respectively. For example, in a test of toddlers’ sensitivity to mispronunciations of tones, Chinese toddlers were very sensitive to lexical tones in a word recognition paradigm (Singh et al., 2015). Similarly, in English infants, Curtin (2010) demonstrated that infants are very sensitive to lexical stress when learning new words. Placed in the current context, it might be the case that the joint skill in phonological encoding of lexical tones and lexical stress underlies the direct contribution of Cantonese lexical tone sensitivity to English lexical stress sensitivity. Speculatively, ours results might be taken to imply that English lexical stress and Cantonese lexical tone were not entirely separate representations, either in the case that L2 English lexical stress was assimilated to L1 Cantonese lexical tone categories (Luke, 2000; Lai, 2003; Chan, 2007), or that L2 English lexical stress and L1 Cantonese lexical tones shared a common “general suprasegmental prototype” as posited by Tong et al. (2015a). These claims were not directly evaluated in the current study, and await further evidence.

### Indirect Contribution from Cantonese Lexical Tone Sensitivity to English Lexical Stress Sensitivity: General Auditory Sensitivity as a Mediator

The indirect pathway through which Cantonese lexical tone sensitivity contributed to English lexical stress sensitivity was via the effects of general auditory sensitivity. In line with previous psychoacoustic studies of English lexical stress perception by tonal listeners (Nguyen et al., 2008; Wang, 2008; Yu and Andruski, 2010), the present results suggest that the contribution of Cantonese lexical tone sensitivity to English lexical stress sensitivity was not driven solely by the phonological interpretation of lexical tone/stress but also by a more general perceptual sensitivity to acoustic-phonetic variation. Empirically, our finding has extended previous attested links between general auditory sensitivity and Cantonese lexical tone sensitivity (Zhang and McBride-Chang, 2010, 2014), by further identifying a link between general auditory sensitivity to English lexical stress sensitivity, in part through which Cantonese lexical tone sensitivity contributed to English lexical stress sensitivity. As mentioned previously, lexical tones and lexical stress share at least one common acoustic cue, most notably, fundamental frequency (f0). Given the prominent role of pitch in tone perception (e.g., Gandour, 1981, 1983; Khouw and Ciocca, 2007; Tong et al., 2014) and in stress perception (Yu and Andruski, 2010), it stands to reason that listeners’ sensitivity to lexical tones and lexical stress may depend on their sensitivity to acoustic pitch. In particular, general auditory sensitivity might be a common construct engaged in the perception of lexical tones and lexical stress at lower auditory levels of speech perception articulated in theoretical models of speech perception (e.g., see McMurray et al., 2011 for C-CuRE Model; Tong et al., 2014 for TTRACE Model; Choi et al., 2017 for TTRACE+ Model).

### Relative Contributions of the Direct versus Indirect Pathways

With regard to the relative contributions of the direct and indirect pathways, the current results suggest that the contribution of Cantonese lexical tone sensitivity to English lexical stress sensitivity was more strongly associated with a direct relationship than an indirect relationship. This suggests that the relationship between suprasegmental perception between languages is more heavily influenced by phonological factors than by sensitivity to acoustic-phonetic variation. In terms of the nature of test stimuli, the Cantonese tone discrimination task and English stress perception task both involved the use of real and frequent words. However, the beat perception in music task involved non-speech tones, which did not resemble familiar contours for tones or stress and were thus linguistically irrelevant. We hypothesize that the similarity between tone and stress in terms of structural properties and communicative functions may predispose these cues to shared processing mechanisms.

### Theoretical and Practical Implications

In terms of theoretical significance, the present findings inform the literature the pathways through which L1 phonological skill contributes to L2 phonological skill at the suprasegmental dimension. These findings have practical significance for L2 learners: one might imagine the presence of two similar, but distinct, sources of phonological variation across languages such as tone and stress to cause confusion for L2 learners. Together with previous studies (e.g., Tong et al., 2015a, 2016), the present findings suggest that instead, Cantonese ESL children may harness their sensitivities to lexical tone in the service of lexical stress perception. In particular, the results suggest that L1 Cantonese lexical tone sensitivity contributes to the development of L2 English lexical stress sensitivity directly and indirectly through the mediation of general auditory sensitivity. It is therefore conceivable that improving lexical tone sensitivity might bolster sensitivity to lexical stress on account of evidence of transfer from the present study. Taken a step further, it is also possible that English L1 bilingual learners might benefit from mastering contrastive stress forms in order to enhance their understanding of the Cantonese tone inventory. This question was not tested in the current study and there is a need for future research to explore this research question.

Despite the implications, it should be noted that the present study is a cross-sectional design, which limits the causal inference. Thus, longitudinal data are needed to study the developmental changes of the three metalinguistic skills tested herein, i.e., Cantonese lexical tone sensitivity, English lexical stress sensitivity and general auditory sensitivity. This may help delineate causality among the three variables. Apart from what was suggested in this study, it might also be possible that children with better ability to segregate different auditory pitch have higher potentials of acquiring both lexical tone and lexical stress. The above claims can be evaluated by undergoing cross-lag modeling, which requires a longitudinal design. Additionally, in the current study, only perception tasks were adopted to evaluate children’s sensitivity to lexical tones and lexical stress. Production tasks of lexical tone and lexical stress may give a more complete picture regarding the developmental changes of lexical tone and lexical stress sensitivities among Cantonese ESL children. Similarly, multiple tasks can be adopted in measuring general auditory sensitivity and working memory, such as including pitch interval discrimination task and visual working memory task. Also, future studies may seek to establish associations or dissociations between the neural perceptual mechanisms underlying lexical tone, lexical stress and other prosodic information such as intonation (Gandour et al., 2003), and how they might be shaped by language experience (Xu et al., 2006).

## Conclusion

The current study has gone beyond identifying the contribution of Cantonese lexical tone sensitivity to English lexical stress sensitivity among Cantonese ESL children, and further explored the pathways underlying the contribution. Results suggest that although lexical stress and lexical tone are distinct sources of variation that are used dissociatively in English and Cantonese, sensitivity to these properties of language develops interdependently in Cantonese children who learn English as L2. Our findings suggest that children may be able to harness their sensitivity to suprasegmental phonology of their L1 to efficiently process different sources of suprasegmental phonology in their L2. It is possible that a L2 learner’s ability to detect and draw on cross-language commonalities may be a fundamental principle of learning that stimulates the growth of knowledge in both languages.

## Ethics Statement

We obtained ethical approval from The University of Hong Kong, Education Faculty Research Ethics Committee. Written consent forms were obtained from school principals, parents and the students prior to testing.

## Author Contributions

All authors listed, have made substantial, direct and intellectual contribution to the work, and approved it for publication.

## Conflict of Interest Statement

The authors declare that the research was conducted in the absence of any commercial or financial relationships that could be construed as a potential conflict of interest. The reviewer FEM and the handling Editor declared their shared affiliation, and the handling Editor states that the process nevertheless met the standards of a fair and objective review.
